# New Mechanism for Voltage Induced Charge Movement Revealed in GPCRs - Theory and Experiments

**DOI:** 10.1371/journal.pone.0008752

**Published:** 2010-01-22

**Authors:** Assaf Zohar, Noa Dekel, Boris Rubinsky, Hanna Parnas

**Affiliations:** 1 Department of Neurobiology, Hebrew University, Jerusalem, Israel; 2 School of Computer Science and Engineering, Center for Bioengineering in the Service of Humanity and Society, Hebrew University, Jerusalem, Israel; Memorial Sloan Kettering Cancer Center, United States of America

## Abstract

Depolarization induced charge movement associated currents, analogous to gating currents in channels, were recently demonstrated in G-protein coupled receptors (GPCRs), and were found to affect the receptor's Agonist binding Affinity, hence denoted AA-currents. Here we study, employing a combined theoretical-experimental approach, the properties of the AA-currents using the m2-muscarinic receptor (m2R) as a case study. We found that the AA-currents are characterized by a “bump”, a distinct rise followed by a slow decline, which appears both in the On and the Off responses. The cumulative features implied a directional behavior of the AA-currents. This forced us to abandon the classical chemical reaction type of models and develop instead a model that includes anisotropic processes, thus producing directionality. This model fitted well the experimental data. Our main findings are that the AA-currents include two components. One is extremely fast, 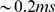, at all voltages. The other is slow, 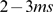 at all voltages. Surprisingly, the slow component includes a process which strongly depends on voltage and can be as fast as 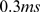 at 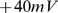. The reason that it does not affect the overall time constant of the slow component is that it carries very little charge. The two fast processes are suitable candidates to link between charge movement and agonist binding affinity under physiological conditions.

## Introduction

Voltage gated channels were shown to exhibit charge movement associated currents, gating currents (GCs), already more than 30 years ago [1]. Since then, an overwhelming amount of experimental data was accumulated [2]–[6]. This data supplemented by mathematical models [7]–[10] indicates that voltage induced reorientation of electric charge within the channel protein produces a conformational change in the protein which leads to channel opening.

G-protein coupled receptors (GPCRs) are the largest family of proteins in the living cell and they mediate most signal transduction processes; the first step being binding of an agonist. Although being transmembrane proteins, they were not considered to be able to sense changes in membrane potential. Recently, however, it was found that several GPCRs exhibit voltage sensitivity where voltage modulates their agonist binding affinity [11], [12]. Even more dramatic was the finding that GPCRs, like channels, display depolarization induced charge movement associated currents. Furthermore, a tight correlation was found between the charge that moves and the fraction of receptors that undergo a change in binding affinity at any membrane potential [13]. Because in GPCRs, the charge movement associated currents lead to alteration of the Agonist binding Affinity we denote these currents AA-currents.

Aiming at unraveling the mechanism that underlies the voltage induced change in agonist binding affinity we developed here a combined theoretical-experimental approach to study the properties of the putative voltage sensor(s), taking the m2-muscarinic receptor (m2R), a prototypical GPCR, as a cases study. We found that the AA-currents include two components. One is in the tenth of millisecond range and the other is slow, 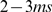. However, the slow component includes a fast constituent which is also in the tenth of millisecond range and carries very little charge. The two fast constituents are suitable candidates to link between charge movement and agonist binding affinity under physiological conditions.

## Materials and Methods

### Preparation of cRNA and Oocytes

cDNA plasmid of m2R was linearized and transcribed in vitro as described [14]. *Xenopus* oocytes were isolated and incubated in NDE solution composed of ND96 (in 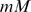: 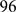 NaCl, 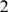 KCl, 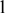 CaCl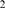, 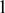 MgCl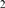, 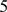 HEPES-NaOH pH 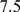) with the addition of 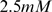 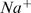 pyruvate, 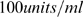 penicillin, and 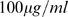 streptomycin. A day after their isolation, the oocytes were injected with 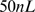 of the m2R cRNA, at 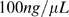. The oocytes were maintained at 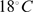 in NDE for 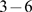 days before currents measurements.

### Cut-Open Oocyte Voltage Clamp Setup

AA-currents recordings were performed with a CA-1B amplifier (Dagan, Minneapolis), as described [13], [15]. Voltage commands were generated by using PCLAMP8 software (Axon Instruments, Union City, CA), a personal computer and a DigiData 1322A interface (Axon Instruments). Recording were performed at room temperature, unless specified otherwise. Data was sampled at 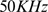 and filtered at 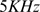. Linear leak and capacitive currents were compensated by analog circuitry and online subtraction employing the 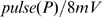 protocol from a holding potential of 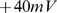 [16]. That is, administrating 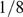 of the test pulse amplitude from a holding potential where no charge movement was observed (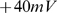). Then, subtracting the currents obtained from 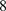 such pulses from the test pulse current. The external solution contained (in 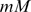) 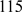 N-methyl-D glucamine (NMDG)-methanesulphonate (NMG-MES), 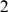 CaCl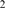, 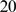 HEPES, pH 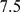. The internal solution was similar but did not contain CaCl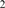 and contained 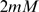 EGTA.

### Statistical Evaluation

Significance was checked by Student's two-tailed or one-tailed t test. Results are given as mean 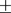 SD.

### Numerical Simulations and Parameters Estimation

All the numerical operations were done using Matlab 7. The equations of the kinetic models were implemented in Matlab and were solved using stiff differential equations variable order method. Fitting eqs. 13 and 14 to the experimental AA-currents recordings was done using the curve fitting toolbox. Parameters optimization was done using multidimensional unconstrained nonlinear minimization method.

## Results

### Characteristic Features of the AA-Currents

**Figure 1:**
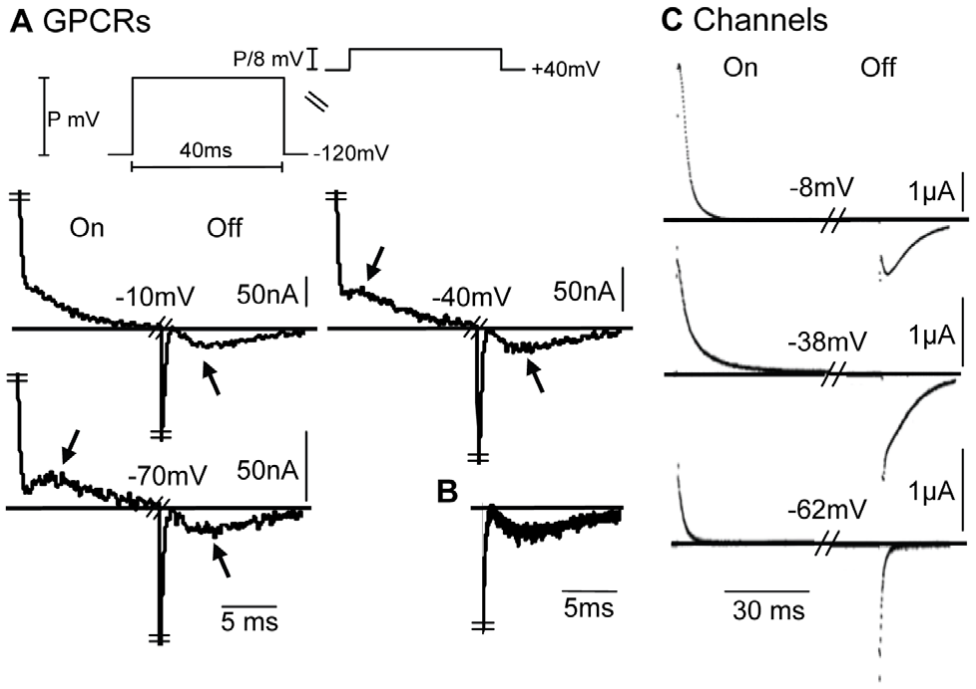
Characteristic features of AA-currents and GCs. (A) AA-currents recorded from m2R expressing oocytes following 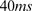 depolarizing pulses to the indicated potentials from a holding potential of 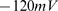, notice the different scales. The arrows in (A) indicate the bumps observed in the AA-currents. Upper panel - the experimental protocol. Symmetric capacitive currents were subtracted by using pulses of 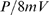 from a holding potential of 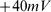. (B) Superposition of the results in (A) where each graph is normalized to the peak amplitude of its fast component. (C) recordings of GCs from oocytes expressing the Shaker 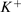 channel, taken with permission from Bezanilla et al. [7], notice the different scales.

Using the cut-open oocyte voltage clamp setup [15], we measured AA-currents in m2R expressing *Xenopus* oocytes. As seen, for the experimental protocol exhibited in Figure 1A (administrating 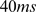 depolarizing pulses of various amplitudes from a holding potential of 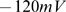, denoted standard protocol), the m2R AA-currents exhibit the following characteristic features: 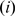 The predominant feature is the “bump”, a complex behavior of a rather fast rise followed by a slow decay which appears both in the On and the Off responses. When existing it always follows the initial fast decay. 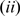 In the On responses, the bump is clear at 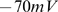, less apparent at 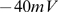 and completely disappears at more positive potentials. 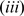 In the Off responses, the bump always appears irrespective of the level of the depolarizing pulse. Furthermore, the normalized responses overlap (Figure 1B). For comparison, the GCs in channels (Figure 1C) show a fundamental different behavior. Specifically, the bump does not characterize the GCs, it rather seems as an odd phenomenon appearing only at very specific conditions; in the Off response of 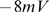 (Figure 1C). Another important difference is that, in contrast to the AA-currents, the Off responses do not overlap. Rather, the behavior of the Off responses depend on the level of the depolarizing pulse.

**Figure 2:**
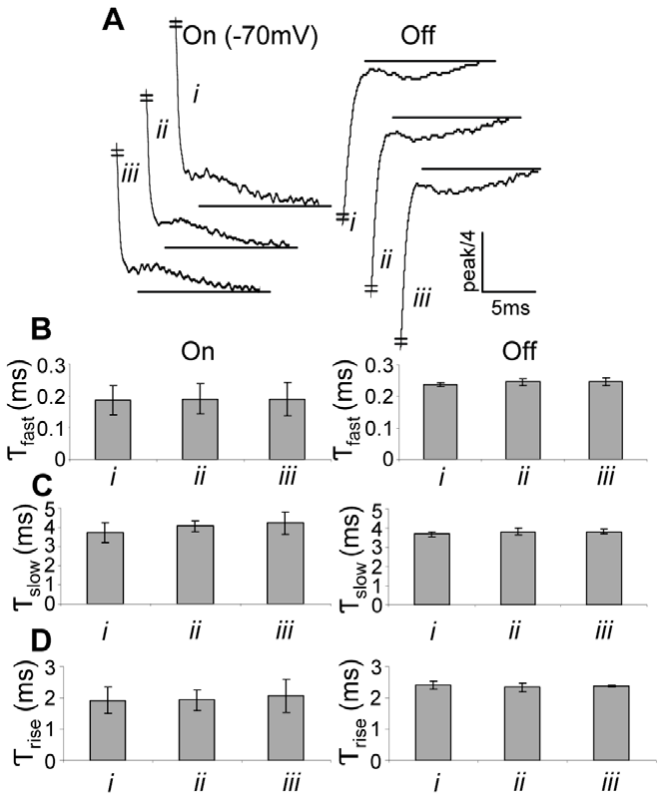
The effect of the subtraction holding potential on the AA-currents kinetics. The AA-currents were subtracted by using 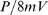 from a holding potential of 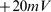 (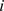), 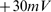 (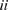) and 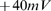 (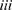). (A) Left panel, On currents elicited following 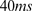 depolarizing pulse from 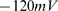 to 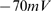. Right panel, Off currents elicited in the return to the holding potential (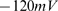). The graphs are normalized, each to the peak amplitude of its fast component. (B) Time constants of the fast component of the AA-currents. (C) Time constants of the slow component of the AA-currents. (D) Time constants of the bump rising phase. The results in B, C and D are presented as mean 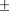
SD (n = 5–60).

Because the bump is a predominant feature of the AA-currents, we designed experiments to check whether the bump is not an artifact but rather an intrinsic feature of the AA-currents. The AA-currents seen in Figure 1A were obtained by subtracting the linear capacitive currents from the total currents [15]. It is, however, possible that residual AA-currents still occur at the voltages used to asses the linear capacitive currents, hence an artificial bump is seen. To check for this possibility, we employed the 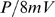 subtraction protocol from three holding potentials (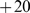, 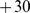 and 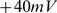), all at the saturated region of the curve that describes the dependency of the charge that moves on depolarization (see later ). Figure 2A shows that the kinetics of the AA-currents (normalized each to the peak amplitude of its fast component) is similar in the three subtraction holding potentials. In particular, the time constants of the fast decay, the slow decay and the rise of the bump obtained by fitting the AA-currents to a three exponential function, are not much affected by the subtraction holding potential (Figure 2B–D). Employing even more positive potentials for subtraction is problematic as it leads to opening of intrinsic voltage-dependent 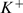 channels [17], [18]. Using holding potentials that are more negative than 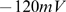 are also problematic because of hyperpolarization-activated 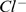 channels [18], [19]. The quantitative features of the bump are not affected even if the only ions existing in the recording system, i.e., 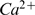 and 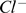, are replaced by 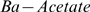 (Figure S1).

**Figure 3:**
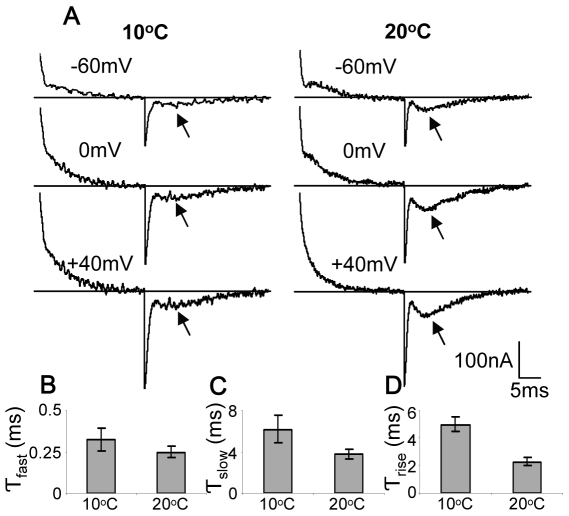
Effect of temperature on AA-currents kinetics. (A) Currents recorded at two temperatures, 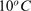, left panel and 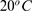, right panel following 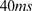 depolarizing pulses to the indicated potentials from 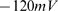. Arrows indicate the bump in the Off responses. B–D, time-constants of various features of the Off responses. (B) Time constants of the fast component. (C) Time constants of the slow component. (D) Time constants of the bump rising phase. The results in B, C and D are presented as mean 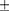
SD (n = 36).

To further challenge our conclusion that the observed bump is an intrinsic feature of the AA-currents, we examined the dependency of the AA-currents kinetics on temperature. We focused on the Off responses where the bump always occurs. We expect that the 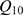 of the various time constants of the AA-currents, including that of the bump, will be in the range previously found for GCs in channels, i.e., 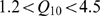 [20]. Figure 3A shows representative AA-currents recorded at 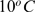 and 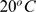. As seen, the Off bump flattened at 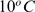 compared with 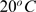. Average of 36 recordings showed that the bump amplitude was reduced by 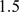 fold (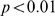) and the average time to peak of the bump was prolonged from 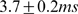 to 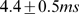 (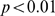) when the temperature was reduced to 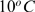. The various time constants of the Off responses (Figure 3B–D) were calculated by fitting the currents to a three exponential function. The time constants of the fast decay, the slow decay and the rise of the bump were increased by 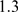, 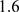 and 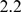 fold respectively when the temperature was reduced to 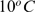 (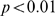).

### Guide Lines in Developing a Mathematical Model for the AA-Currents

To obtain a bump, a minimum of two sequential transitions is required [21]. Accordingly, a minimal model that will produce a bump will be,



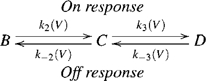
(1)


For the model in scheme 1 to actually produce a bump two additional requirements must be met. (a) the parameters should guarantee that the 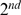 transition (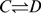 in the On response and 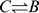 in the Off response) will produce most of the measured AA-currents. For further details and means to achieve (a) see Text S1, Eqn S6. (b) Before administration of the depolarizing pulse the receptors need to populate mainly state 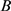 while at the end of the depolarizing pulse the receptors need to populate mainly state 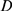. This guarantees a delay in populating state 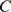.

In order to account for the fast component seen in Figure 1A, an additional transition needs to be added either sequentially (scheme 2) or in parallel (scheme 3).



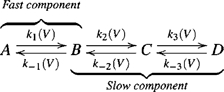
(2)


Scheme 2 can account for feature (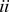) and partially for feature 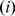, but fails to produce feature (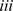) of the AA-currents(see Figure S2A). The failure to produce feature (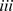) stems from the following. The Off response depends heavily on the occupancies of the various states at the end of the depolarizing pulse. In particular, following a high pulse, mainly state 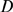 will be populated. Hence, the Off response will begin with the slow transition 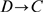 and will lack the initial fast component. In contrast, following a low pulse mainly states 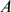 and 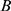 will be populated. Hence, the Off response will show a fast decay but will lack a bump altogether. Depending on the pulse amplitude we will observe behaviors which vary between the two extreme cases, as seen for channels (Figure 1C).

To ensure that the Off response will always exhibit an initial fast component independently of the pulse amplitude we modified the sequential model (scheme 2) to become a parallel model (scheme 3). Here, the fast component occurs in parallel to the slow one.



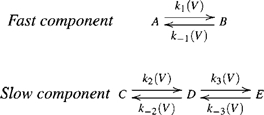
(3)


Indeed, this model accounts for the fast decay that precedes the bump in the Off responses (part of feature (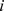)). However, similar to the model in scheme 2, it fails to consistently produce a bump in the Off response (Figure S2B).

An additional model that was designed to produce a rising phase, hence a bump, is a pair of interacting charges that undergo spatial diffusion along a bi-stable potential of mean force [22]. This model was simplified to a 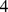 state cyclic Markovian model as depicted in scheme 4 where we assign similar symbols as before to the various states. Without loss of generality we assume that during the On response the receptors shift from state 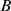 to state 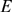 while the opposite occurs during the Off response.



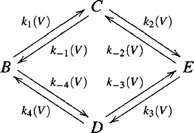
(4)


However, such a model, under the constraint of microscopic reversibility, can produce a prominent bump but only either in the On or the Off responses (Figure S3). Hence it cannot account for feature 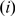 characterizing the AA-currents.

### The AA-Current Model

What do we learn from the former models? Because the parallel model (scheme 3), but not the sequential one (scheme 2), accounts for an initial fast component followed by a slow one both in the On and the Off responses we retain in the final model the property that the fast component occurs in parallel to the slow one. Regarding the slow component, it must involve two transitions occurring in sequence in order to produce a bump. But, because the sequential part of the previous schemes does not guarantee a consistent depolarization independent bump in the Off responses the two transitions must occur in parallel rather than sequentially. Reconciliation of the two contradicting requirements; i.e., the two transitions occurring in parallel and in sequence, is achieved as follows. The slow component is now composed of two parallel transitions. The first transition, 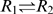, is faster than the second transition 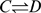. The second transition is coupled to the first one by its voltage dependent rate constants being also dependent on the receptor configuration, 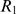 or 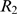. Such a model generates directionality.

The final model of the AA-currents is presented in scheme 5 and Eqn 6.



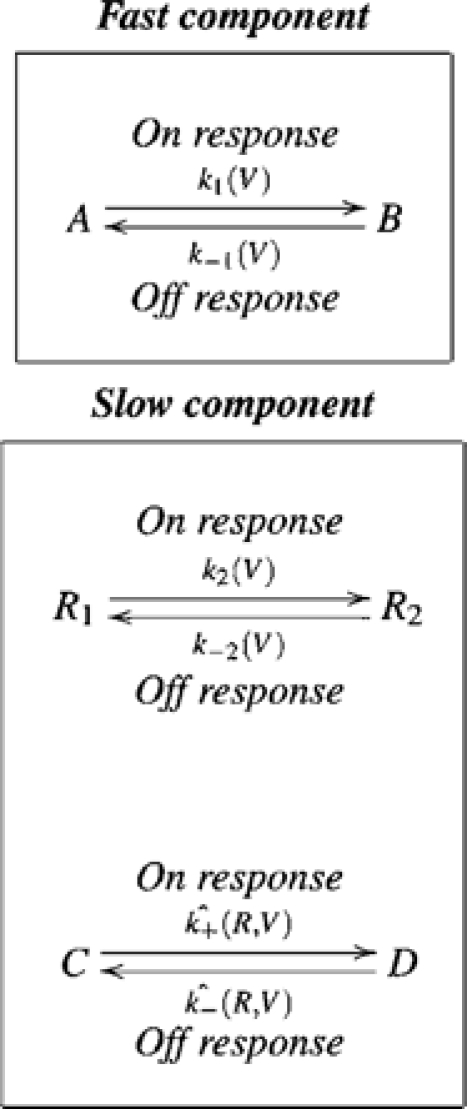
(5)


To ensure that the transition 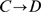 (On response) will indeed follow the transition 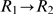, the rate constant for the transition 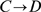 must be small when the receptor is in 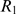 and large when in 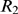. Similarly, to ensure that the transition 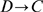 (Off response) will indeed follow the transition 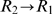 the rate constant for the transition 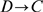 must be small when the receptor is in 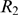 and large when in 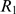. Accordingly,



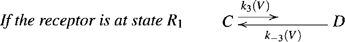
(6a)




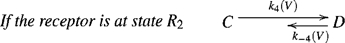
(6b)


To obtain 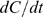 we need to provide a quantitative description for 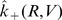 and 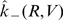. Following scheme 5 we notice that the receptor can be in one of four complex states; 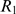 and 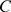; 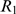 and 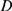; 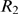 and 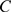 and 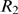 and 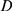. Thus, the fraction of receptors that will transit from state 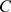 to 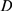 with the rate constant 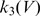 (Eqn 6a) will be the fraction of receptors that are in the complex state of 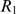 and 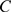. Because the 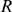 states are independent of state 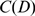 (scheme 5), this fraction equals 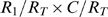, where 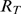 is the amount of total receptors. To transform from fractions to amount of receptors the fraction of receptors at the complex states needs to be multiplied by 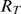. Accordingly,
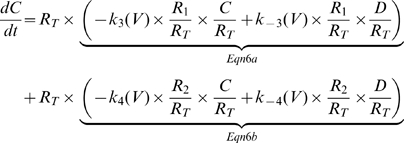
(7)


Rearrangement of Eqn 7 provides,
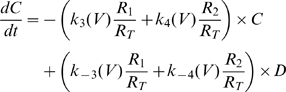
(8)


Recalling that the transitions 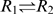 do not depend on whether the receptor is in state 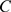 or 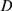, we obtain,
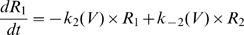
(9)


For completion of the model equations, Eqn 10 describes the fast component,
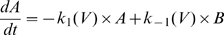
(10)


The model differential equations obey the following conservation law,
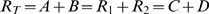
(11)


Thus, the AA-currents model is given by Eqn 8–11. And the total AA-currents (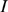) are given by the sum of the effective charges (the valence of the charge times the fraction of the electric field it traverse) carried by transitions 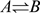, 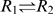 and 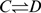. Thus,
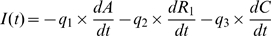
(12)


Where 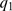, 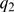 and 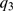 are the effective charges carried by transitions 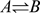, 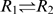 and 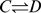 respectively.

### Parameters Estimation

To obtain an initial estimation of the various rate constants we aimed at achieving an analytical solution for the AA-currents. This became possible by simplifying scheme 5. Based on Eqn 6 we neglected 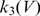 and 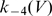. Furthermore, because 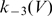 decreases with depolarization and increases with hyperpolarization while the opposite occurs for 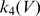 we neglected 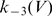 and 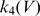 during the On and Off responses respectively. The analytical solution is given by (for further details see Text S1, Eqn S17–S20),
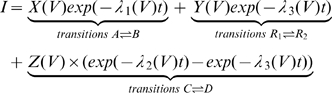
(13)


Where 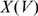 is composed of the effective charge carried by transitions 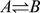 and of the rate constants of these transitions. 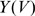 is composed of the effective charge carried by transitions 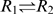 and of the rate constants of these transitions. 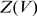 is composed of the effective charge carried by transitions 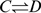 and of the rate constants of transitions 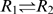 and 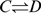. The exponents 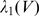, 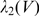 and 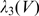 are defined as the sums 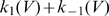, 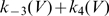 and 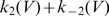 respectively (for further details see Text S1, Eqn S19).

The solution of Eqn 13 showed good agreement with the experimental results of Figure 1A (Figure S4A). It is satisfying to note that the parameters estimated from Eqn 13 corroborate the requirement of condition (a) above, necessary for a bump to be produced, that the contribution of transitions 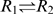 to the AA-currents is negligible, i.e., it is a “hidden” transition (see Figure S4B). Neglecting this charge, Eqn 13 is further simplified to become,
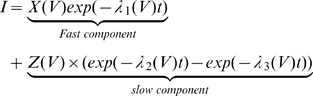
(14)


The initial set of parameters was obtained by fitting Eqn 14 to the experimental results. The rate constants were estimated from the corresponding exponents in Eqn 14, i.e., 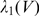, 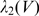 and 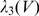, employing exponential voltage dependency as described in Bezanilla et al. [7]. The effective charges carried by transitions 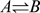 and 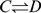 were estimated from 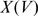 and 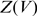 respectively using the already estimated rate constants.

Finally, the full model (scheme 5 and Eqn 6) was solved numerically and the initial values were adjusted (for most parameters no more than 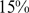 change was required) such as to obtain best fit to the experimental results. Specifically, we used unconstrained nonlinear optimization method using the initial parameters estimation as an initial value. The final values of the parameters are given in Table S1.

### Use of the Model to Characterize the Properties of the AA-Currents

**Figure 4:**
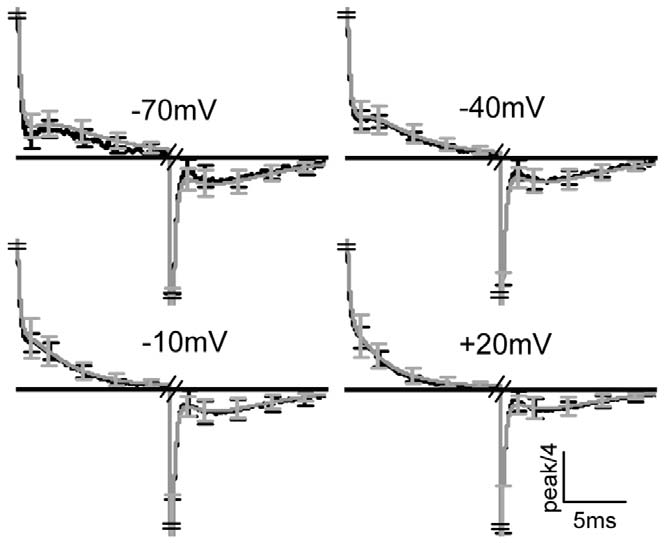
Comparing simulation and experimental results employing the standard protocol (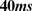 pulse duration, 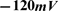 holding potential). Average 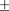
SD (n = 5) currents at indicated depolarizing pulses, model, gray lines, experiments, black lines. The experimental currents are normalized, each to the peak amplitude of its fast component and the simulations are normalized, each to the peak amplitude of the corresponding experimental fast component (see text for details).

We begin by contrasting the model and the estimated parameters (Table S1) with experimental results employing the standard protocol. For both, normalized average results with SD are provided. The normalized average of the simulation results was obtained as follows. Parameters were estimated for each of five individual oocytes. Then, five simulations of the AA-currents corresponding to the five sets of parameters were conducted. Finally each simulation was normalized to the peak amplitude of the fast component of its corresponding experimental result. This was done to contrast both the simulations kinetics and amplitude with the experimental currents. Then, as for the experiments, the average of the simulation results was found. Figure 4 shows that the simulations (gray lines) and the experiments (black lines) match very well both in the On and the Off responses. Also seen that the model successfully accounts for the characteristic features of the AA-currents (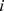
–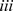 above).

**Figure 5:**
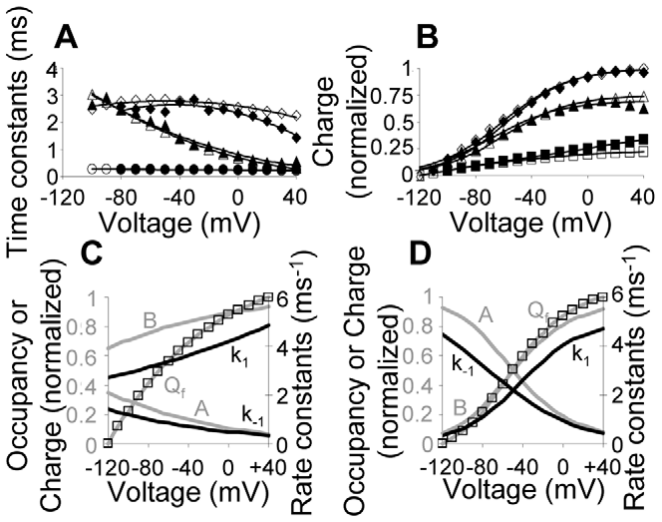
Comparing average (n = 5) simulation and experimental results employing the standard protocol. (A) Time constants of the various transitions, 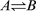 (circles), 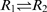 (triangles) and 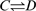 (diamonds). Here and below, open symbols correspond to the model and filled symbols to the experiments. (B) 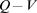 curves of the total charge (diamonds), the fast component (squares) and the slow component (triangles). (C) and (D) Average occupancies of the fast component states 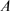 and 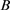 (gray lines), the values of 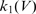 and 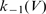 (black lines) and the simulated normalized charge carried by the fast component (squares). In (C) the rate constants were estimated under the constraint of exponential dependency on voltage [7], while in (D) the rate constants were estimated after relaxing this constraint (Table S1 and Table S2 respectively).

To further characterize the behavior of the AA-currents we need to estimate the time-constants of each component and the dependency of the charge that moves by each component on voltage (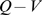). A straightforward approach to evaluate the experimental time-constants would be by fitting the results to a double exponential decay function [7]. This procedure is not efficient in the present case due to the existence of a prominent bump. Because Eqn 14, a sum of three exponents, was shown to faithfully describe the experimental results (Figure S4C), we evaluated the time-constants of the fast component and that of the two constituents of the slow component from this equation. Figure 5A shows that the time-constant of the fast component (filled circles) is around 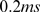 and depends only weakly on voltage. The time-constant of the slow constituent of the slow component (filled diamonds) exhibits a parabolic dependency on voltage with values ranging between 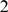 to 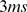. The surprising behavior concerns the time-constant of the other constituent of the slow component (filled triangles); It exhibits strong voltage dependency ranging from 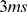 at 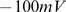 to around 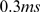 at 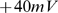.

The time-constants of the various components can be also evaluated from the model. Here, the time-constant of the fast component corresponds to transitions 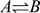. The time-constant of the slow constituent of the slow component corresponds to transitions 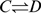 and the fast constituent of the slow component corresponds to transitions 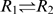. It can be seen that the time-constants evaluated from the model (Figure 5A, open symbols) match well the experimental ones.

Because transitions 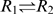 carry insignificant fraction of the charge, these transitions cannot be detected experimentally. Incorporating transitions 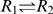 into the model was necessary for generation of the directionality required to account for the bump observed in the On and the Off responses. It is thus important to examine whether the characteristic properties of the transitions 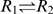 (insignificant charge and strong voltage dependency) are indeed essential. We show in Figure S5 that if these transitions carry charge larger than 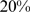 of the charge carried by transitions 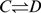 or if they show weak voltage dependency the model fails to show a bump.

To evaluate how much charge is carried by each component we show in Figure 5B their respective 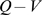 curves. We see that as for the kinetics, the model also describes well the steady-state features of the AA-currents. The total charge (integral of the total current) shows a characteristic sigmoid shape. Also seen (extracted from Eqn 14) is that the slow component carries most of the charge (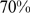). The 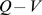 curve of the fast component, which is controlled by the transition 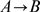, lacks the characteristic initial plateau (Figure 5C, squares), implying that a significant fraction of 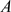 shifted to 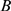 already at voltages lower than 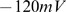, which could imply that this transition is physiologically irrelevant. Indeed, when 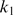 and 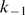 depend exponentially on voltage (Figure 5C, black lines) we find that already at 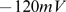 more than 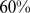 of the receptors are at state 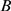 (Figure 5C, gray lines). Due to the physiological importance of this issue and because the estimation of the rate constants was performed with the constraint of exponential voltage dependency [7], we re-estimated the parameters but now relaxing this constraint. The parameters obtained from this fit are summarized in Table S2. Surprisingly, although no constraints were employed, the rate constants 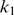 and 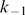 exhibit a sigmoid voltage dependency (Figure 5D, black lines) while the rest of the rate constants maintained the exponential voltage dependency.

Figure S6 depicts a comparison between the experiments and the simulations employing unconstraint parameters. It is seen that the fit to experimental results is even better than the very good fit obtained with the constraint parameters. However, in contrast to the behavior with the constraint parameters, the transitions 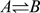 exhibit significant voltage dependency under the physiological range of the action potential (Figure 5D, gray lines). Specifically, at membrane potential of 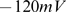 more than 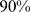 of the receptors are at state 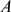 while at membrane potential of 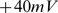 more than 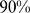 of the receptors are at state 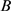. Furthermore, under these conditions the 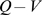 curve of the fast component exhibits the characteristic initial plateau (Figure 5D, squares).

### Further Experimental Validation of Key Assumptions of the Model

So far we have shown that the model fits well various aspects of the experimental results. We, nevertheless, wish to further test the model by examining whether its key assumptions can be validated experimentally. To do so, we first examine how the model generates the characteristic features of the AA-currents.

**Figure 6:**
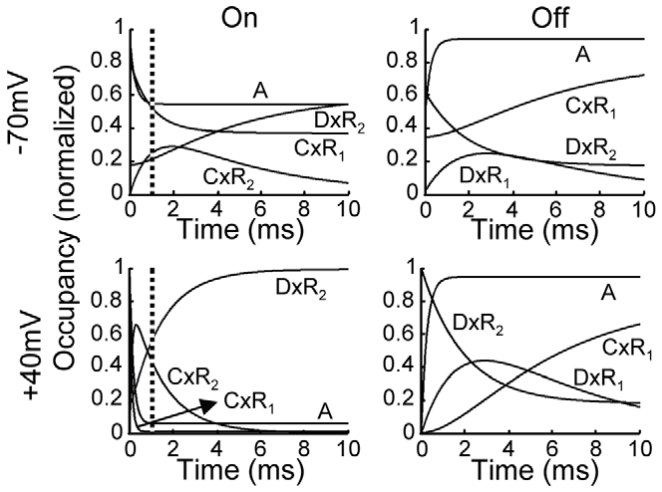
How the model generates the AA-currents features. Occupancies of relevant states of the model during the On (left column) and the Off (right column) responses employing the standard protocol. The simulation results were obtained using the unconstraint parameters (Table S2).

What generates the bumps in the On responses? Recalling Eqn 6, it is the transition 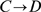 employed with the rate constant 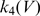 (receptors that are at state 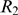) which is expected to form the bump during the On response. Indeed, the occupancy of the receptors that are both at state 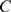 and 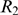 (denoted 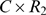) shows a bump like behavior (Figure 6, left panel). Comparing the time to peak of 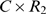 to that of the bump at the same voltage (Figure 4, 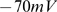) we find that they match. The rising phase of the bump is thus controlled by the transitions 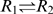.

Why is the bump in the On response detected only at low depolarizations? Examining the time course of the fast transition 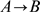 at 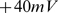 (Figure 6, lower panel) and at 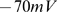 (Figure 6, upper panel), we find that the two are very similar. In contrast the transition 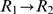 is much faster at 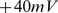 than at 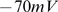 (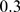 and 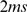 respectively). Therefore, although a bump is formed both at 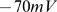 and at 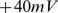, it will be detected only at 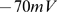 because at 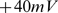 it merges with the fast transition 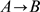.

Why is the bump in the Off response always observed irrespective of the depolarizing pulse? In the Off response it is the occupancy of 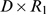 which correlates with the bump (Figure 6, right panel). As seen, the time to peak of the bump, which is governed by the transition 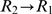, is very similar at both 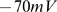 and 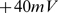. This is because the slow time constant of the transition 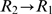 corresponds to 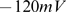 (the holding potential). At the same time, the fast component, whose rate constant also corresponds to 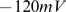, remains fast. Consequently, the bump is distinct from the fast component at all voltages.

**Figure 7:**
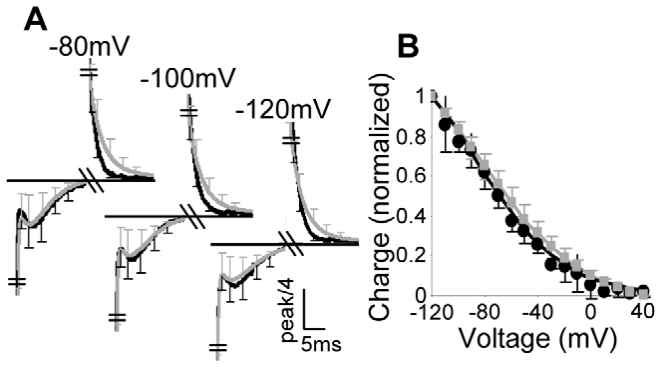
Kinetics of the AA-currents and 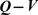 curves employing the reverse protocol. (A) Kinetics of the experimental and simulation results. The currents are normalized as describe in Figure 4. Here and in B, experiments, black lines and simulations gray lines. (B) 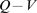 curves of the total charge. Experimental and simulation results are presented as mean 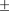
SD (n = 5). The simulation results were obtained using the unconstraint parameters (Table S2).

To test the above conclusion experimentally, we employed a “reverse” protocol. That is, we now administered pulses which are below 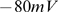 from a holding potential of 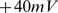. We expect to see a bump during the On response for all these pulses. Upon return to the holding potential of 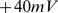 (now the Off response) we expect a double exponential decay. Figure 7A shows that these predictions are fully met. The 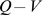 curve of the reverse protocol is given in Figure 7B. It is seen that the model predictions (gray lines) match extremely well both the kinetics (Figure 7A) and the steady-state (Figure 7B) results. Since the model parameters were estimated from the standard protocol, the excellent match between the model and the reverse protocol results provides further support to the model.

**Figure 8:**
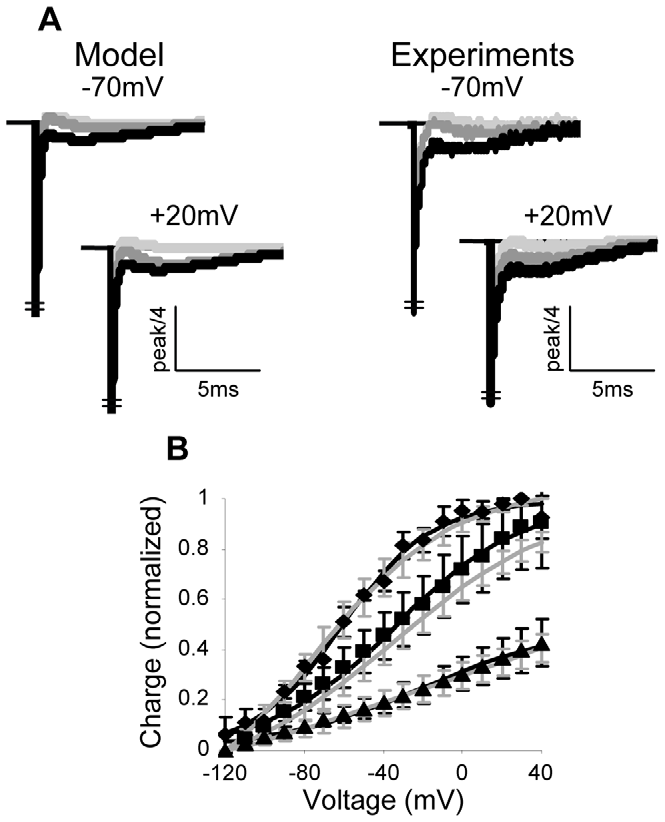
The effect of pulse duration on the characteristics of the AA-currents. (A) Experimental (right panel) and simulation (left panel) results employing the standard protocol with pulse duration of 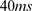 (black lines), 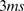 (dark gray lines) and 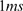 (light gray lines). The currents are normalized as describe in Figure 4. (B) Corresponding 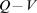 curves of the total charge, pulse durations of 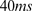 (diamonds), 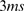 (squares) and 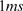 (triangles), experimental (black lines) and model (gray lines). Experimental and average simulation AA-currents are presented as mean (A) or mean 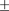
SD (B) (n = 5). The simulation results were obtained using the unconstraint parameters (Table S2).

Providing support to the notion that transitions 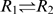 govern the bump, we now test the notion that the rate constants of these transitions depend strongly on voltage. To do so, we will repeat the experiment of Figure 1A but with a brief pulse instead of the standard 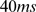 pulse and will focus on the Off response. The rational underlying such experiments is as follows. The Off response will be measured because the bump during the Off response depends on the fraction of receptors that populate states 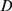 and 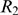 at the end of the On response. We recall that the time constant of the transitions 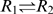 is 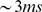 at low depolarizations, but it is 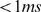 at high depolarizations. Thus, with a 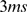 pulse we expect to see almost no bump at low depolarizations and a prominent bump at high ones (Figure 8A, left panel). Figure 8A, right panel, shows that these predictions are fully met by the experimental results. As expected, following a pulse of 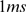 (shorter than the time constant of transitions 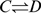) no bump is formed at any voltage. Figure 8B depicts the 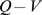 curves of the various pulse durations normalized to that obtained following 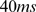 pulse. As seen, the model also accounts for the steady-state behavior of the short pulses. Furthermore, because following the 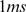 pulse only the fast component occurred, this result further supports the conclusion that the fast component carries only 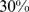 of the charge (Figure 5B).

## Discussion

We examined several models to their ability to account for the main characteristic features of the AA-currents. We found that only a non-linear model with rate constants that guarantee directionality can match the experimental results.

Conventional modeling of chemical reactions employ the Curie theorem which states that forces of one tensorial order cannot couple with fluxes of a different order. In the Curie theorem sense the affinity of a chemical reaction which is a scalar cannot couple with a vector such as the transition of a carrier from one side of the membrane to the other. In that sense a chemical reaction cannot drive a directional process, it can only cause activation or deactivation of a carrier. This concept is also closely tied to the concept of thermodynamic equilibrium. Conventional chemical reactions represent states of thermodynamic equilibrium. On the other hand the flux of a species cannot be, by definition, in a state of thermodynamic equilibrium. Therefore, conventional chemical reaction formulations of equilibrium are not rigorously correct when describing a vectorial process.

In our study we have encountered a situation in which there is a directional flux of electrical charges, i.e., a vectorial process that is not in a state of thermodynamic equilibrium. The bump pattern which we have observed (Figure 1A) involves a process that is clearly not in thermodynamic equilibrium and has directionality. Indeed attempts to employ conventional chemical reaction equilibrium formulations to describe the process failed. Therefore we used a new constitutive type of formulation in which we imposed a vectorial behavior to the process. It has been proposed [23] that anisotropy can act as a universal feature of vectorial couplings, the Curie theorem notwithstanding. We adopted this concept in Eqn 6. We showed that the constitutional anisotropic representation could indeed represent the experimentally observed process. Since the only fundamental thermodynamic criteria that constitutive relations must satisfy are the laws of conservation, we have checked conservation of mass and have shown that the new formulation satisfies conservation of mass (Eqn 11).

Which of the AA-currents components could be responsible for the observed [11]–[13] voltage induced agonist binding affinity change? We previously showed [13], regarding the m2R, a tight correlation between the dependence of the charge that moves on voltage (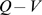) and the dependence of the fraction of receptors in low affinity state on voltage (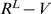), suggesting that it is the charge that moves that drives the change in the GPCR's binding affinity. Under physiological conditions the voltage induced change in binding affinity is expected to be produced by the brief (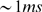) action potential. Indeed this was shown to be the case for release of acetylcholine [24] and glutamate [25] from nerve terminals. Therefore, the two fast components, one from the fast voltage sensor and the hidden one from the slow voltage sensor, exhibit an appropriate time constant (faster than 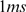). The fast voltage sensor is a natural candidate. This is because we showed here that this fast component contributes 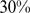 of the total charge that moves. Hence, it could play a major role in relaying charge movement to changes in conformation of the receptor and as a result change in binding affinity. The situation is different regarding the fast component of the slow voltage sensor (the transitions 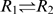), which does not display significant charge movement. Therefore, at first sight, this component is not expected to play a role in relaying charge movement to changes in conformation of the receptor. The question is then whether this fast component, whose rate constants strongly depend on voltage, can nevertheless be responsible for change in binding affinity. We argue that this could well be the case. It had been suggested regarding class A GPCRs (to which the m2R belongs) that a network of salt bridges forms an ionic lock that is disrupted during receptor activation [26]–[30]. It is thus quite possible that the transitions 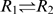 represent a voltage dependent break of a salt bridge, hence the strong voltage dependency of the rate constants. This break, in turn, could cause a chain of conformational changes in the receptor resulting in changes in agonist binding affinity.

Our data is too preliminary to suggest a biophysical mechanism for the charge movement in GPCRs. However, it is possible that the main difference in the kinetics of GCs in channels and AA-currents in GPCRs stems from the lack of voltage dependent break of a salt bridge in the former.

## Supporting Information

Text S1 The supporting information text and equations.
(0.11 MB PDF)

Figure S1 The effect of the ions present in the external solution on the kinetics of the AA-currents. On and Off currents elicited in the m2R expressing oocytes following 40ms depolarizing pulses to the indicated potentials from −120mV. Standard external solution (black lines, see Methods) and when 2mM of CaCl2 was replaced by 2mM of Ba-Acetate (gray lines). The graphs are normalized, each to the peak amplitude of its fast component and are presented as mean ±SD (n = 4–9).
(0.06 MB TIF)

Figure S2 AA-currents predicted by the sequential (scheme 7) and the parallel (scheme 8) models employing the standard protocol to the indicated potentials, (A) and (B), respectively. The Off AA-currents predicted by the slow component of the parallel model (scheme 8) are depicted in the inset of (B). The graphs are normalized, each to the peak amplitude of its fast component. The pulse protocol is presented on top.
(0.13 MB TIF)

Figure S3 AA-currents predicted by the cyclic model (scheme 9). Computed AA-currents employing 40ms depolarizing pulse to −70mV from a holding potential of −120mV. The model was assigned with parameters that satisfy the conditions in Eqs. 15 and 16 and microscopic reversibility. Inset - the initial phase of the Off response.
(0.09 MB TIF)

Figure S4 Fitting of Eq. 20 and 21 to the experimental AA-currents recordings. (A) AA-currents recordings from m2R expressing oocytes employing the standard protocol (black lines) superimposed with the three exponential fitting function, Eq. 20 (gray lines). (B) The total charge (circles) and the charge carried by the transitions A↔B (triangles), R1↔R2 (diamonds) and C↔D (squares). (C) AA-currents recordings from m2R expressing oocytes employing the standard protocol (black lines) superimposed with the three exponential fitting function, Eq. 21 (gray lines). (D) Separate plot of the fast and the slow components, solutions were obtained from the same equation (Eq. 21) that was used to fit the experimental results seen in (C). Black lines represent the fast component (Xexp(−λ_1_t)) and gray lines represent the slow component (Z×(exp(−λ_2_t)−exp(−λ_3_t))).
(0.26 MB TIF)

Figure S5 Examining the behavior of transitions R1↔R2. (A) Predicted AA-currents following 40ms depolarizing pulse to −70mV from holding potential of −120mV. The three simulations differ in the value that was assigned to the effective charge carried by transitions R1↔R2. The values were 0, 20 and 40% of the effective charge carried by the transition C↔D (blue, green and red lines respectively). Here and below, the graphs are normalized each to the peak amplitude of its fast component. (B) Predicted AA-currents following 40ms depolarizing pulse to +20mV from holding potential of −120mV. The three simulations differ in the time constant that was assigned to transition R1↔R2 at +20mV. The time constants were: 0.134ms (the time constant that was used throughout, blue line) and 5 and 10 times slower (green and red lines respectively). (C) Predicted AA-currents following 40ms depolarizing pulse to −70mV from holding potential of −120mV. The three simulations differ in the time constant that was assigned to transition R1↔R2 at −70mV. The time constants were: 1.9ms (the time constant that was used throughout, blue line) and 5 and 10 times faster (green and red lines respectively). The model simulation results were obtained using the unconstraint parameters (Table S2).
(0.06 MB TIF)

Figure S6 Comparing average (n = 5) simulation and experimental results employing the standard protocol. (A) Kinetics of AA-currents, experiments, black lines, simulations, gray lines. The currents are normalized as describe in Fig. 4 (see text). (B) Time constants of transitions A↔B (circles), R1↔R2 (triangles) and C↔D (diamonds). In all, open symbols correspond to simulations while filled symbols correspond to experiments. (C) Q–V curves of the total charge and the fast and slow components. Diamonds, total Q–V, triangles, Q–V of the slow component and squares, Q–V of the fast component. In all, open symbols correspond to the model and filled symbols to experiments. The parameters were estimated after relaxing the constraint of exponential dependency on membrane potential (Table S2).
(0.10 MB TIF)

Table S1 List of the parameters and the standard deviations estimated for the AA-currents model (text, scheme 4).
(0.03 MB PDF)

Table S2 List of the unconstraint parameters and the standard deviations estimated for the AA-currents model (text, scheme 4).
(0.03 MB PDF)
