# Supplementary material for: New Mechanism for Voltage Induced Charge Movement Revealed in GPCRs - Theory and Experiments
Source: PLoS One. 2010 Jan 22;5(1):e8752. doi: 10.1371/journal.pone.0008752 (PMC2809744; doi:10.1371/journal.pone.0008752)
Supplement: Text S1 — The supporting information text and equations. (0.11 MB PDF) [file pone.0008752.s001.pdf]

Supporting Information

New Mechanism for Voltage Induced Charge Movement Revealed in GPCRs - Theory and Experiments

Assaf Zohar[1,#], Noa Dekel[1,#], Boris Rubinsky[2], Hanna Parnas[1,*]
1 Department of Neurobiology, Hebrew University, Jerusalem, Israel
2 School of Computer Science and Engineering/Center for Bioengineering in the Service of Humanity and Society, Hebrew University, Jerusalem, Israel
∗ E-mail: hannap@savion.huji.ac.il
# These authors contributed equally to this work.

Requirements for a bump to be formed

Sequential model

It was previously shown that in order for a bump to be produced a minimum of two sequential transitions are required [1]. Accordingly,

$$\begin{array}{c} \textit{On response} \\ B \underset{k_{-2}(V)}{\overset{k_2(V)}{\rightleftharpoons}} C \underset{k_{-3}(V)}{\overset{k_3(V)}{\rightleftharpoons}} D \\ \textit{Off response} \end{array} \tag{S1}$$

In scheme S1, the transitions $B \rightleftharpoons C$ and $C \rightleftharpoons D$ form the AA-currents, I. Thus,

$$I = -q2 \times \frac{dB}{dt} + q_3 \times \frac{dD}{dt} = q_2 \times (k_2 \times B - k_{-2} \times C) + q_3 \times (k_3 \times C - k_{-3} \times D) \tag{S2}$$

Where q_2 and q_3 are the effective charges of the first and second transitions respectively and $k_{\pm i}$ are the rate constants.

For the model in scheme S1 to actually produce a bump two additional requirements must be met. (a) The parameters should guarantee that the 2nd transition ($C \rightleftharpoons D$ in the On response and $C \rightleftharpoons B$ in the Off response) will produce most of the measured AA-currents. (b) Before administration of the depolarizing pulse the receptors need to populate mainly state B while at the end of the depolarizing pulse the receptors need to populate mainly state D. Under condition (b) two transitions are guaranteed. However, fulfilling requirement (b) while necessary is not sufficient for a bump to be formed, condition (a) must also be met. We show below how this can be achieved.

Solving the model of scheme S1 under the assumption that before depolarization only state B is occupied while at the end of the pulse only state D is occupied and introducing into Eqn S2 we get,

$$I_{On} = X \times (exp(-\lambda_1 t) + exp(-\lambda_2 t)) + Y \times (exp(-\lambda_1 t) - exp(-\lambda_2 t)) \tag{S3}$$

Where
$X = \frac{k_2}{2} \times q_2 \times R_T$
$Y = \frac{k_2 \times k_3}{I^{1/2}} \times q_3 \times R_T - \frac{k_2}{2I^{1/2}} \times (k_2 + k_{-2} + k_3 + k_{-3}) \times q_2 \times R_T$
$\lambda_1 = \frac{1}{2}(k_2 + k_{-2} + k_3 + k_{-3} - I^{1/2})$
$\lambda_2 = \frac{1}{2}(k_2 + k_{-2} + k_3 + k_{-3} + I^{1/2})$
$I = k_2^2 + k_{-2}^2 + k_3^2 + k_{-3}^2 + 2(k_2 \times k_{-2} - k_2 \times k_3 - k_2 \times k_{-3} + k_{-2} \times k_3 - k_{-2} \times k_{-3} + k_3 \times k_{-3})$

and R_T is the total receptors concentration.

An analogous expression can be obtained for the Off response.

In order to obtain the rising phase of the bump the derivative of the AA-currents at $t = 0$ ($\dot{I}(t = 0)$) needs to be positive. Accordingly,

$$\dot{I}_{On}(t = 0) = -\lambda_1 \times (X + Y) + \lambda_2 \times (Y - X) > 0 \quad \text{(S4)}$$

Rearrangement of Eqn S4 provides

$$\frac{\lambda_1}{\lambda_2} < \frac{Y - X}{Y + X} \quad \text{(S5)}$$

Solving the inequality of Eqn S5 shows that a rising phase will occur only if the model parameters satisfy

$$\begin{array}{ll} \frac{q_2}{q_2+q_3} < \frac{k_3(V)}{(k_2(V)+k_{-2}(V)+k_3(V))} & \textit{during the On response} \\ \frac{q_3}{q_2+q_3} < \frac{k_{-2}(V)}{(k_{-2}(V)+k_3(V)+k_{-3}(V))} & \textit{during the Off response} \end{array} \quad \text{(S6)}$$

The inequality of Eqn S6 for the On response can be achieved if $q_2 \ll q_3$, thus $\frac{q_2}{q_2+q_3} \ll 1$, or if $k_3(V) \gg k_2(V) + k_{-2}(V)$, thus $\frac{k_3(V)}{(k_2(V)+k_{-2}(V)+k_3(V))}$ approaches 1. In both cases the second transition will produce most of the current, hence condition (a) is met. Similar considerations apply also for the Off response.

Requirements for an initial fast decay followed by a bump

As was shown (text, Figure 1A) employing the standard experimental protocol (administrating 40*ms* depolarizing pulses of various amplitudes from a holding potential of $-120mV$), the m2-muscarinic receptor (m2R) AA-currents exhibit three characteristic features. (*i*) The predominant feature is the ”bump”, a complex behavior of a rather fast rise followed by a slow decay which appears both in the On and the Off responses. When existing it always follows the initial fast decay. (*ii*) In the On responses, the bump is clear at $-70mV$, less apparent at $-40mV$ and completely disappears at more positive potentials. (*iii*) In the Off responses, the bump always appears irrespective of the level of the depolarizing pulse. Furthermore, the normalized responses overlap

To account for the initial fast decay (part of feature (*i*)) an additional transition is required. This transition can be added in sequence (scheme S7) or in parallel (scheme S8).

$$\overbrace{A \underset{k_{-1}(V)}{\overset{k_1(V)}{\rightleftharpoons}} B}^{\textit{Fast component}} \underbrace{\underset{k_{-2}(V)}{\overset{k_2(V)}{\rightleftharpoons}} C \underset{k_{-3}(V)}{\overset{k_3(V)}{\rightleftharpoons}} D}_{\textit{Slow component}} \quad \text{(S7)}$$

$$\begin{array}{ll} \textit{Fast component} & A \underset{k_{-1}(V)}{\overset{k_1(V)}{\rightleftharpoons}} B \\ \textit{Slow component} & C \underset{k_{-2}(V)}{\overset{k_2(V)}{\rightleftharpoons}} D \underset{k_{-3}(V)}{\overset{k_3(V)}{\rightleftharpoons}} E \end{array} \quad \text{(S8)}$$

Figure S2 depicts the On and Off AA-currents predicted by the sequential (scheme S7) and the parallel (scheme S8) models. As seen, the sequential model accounts for an initial fast decay followed by a bump in the On response (features (*ii*) and part of (*i*)), but fails to exhibit the same behavior in the Off response (features (*iii*) and part of (*i*)) (Figure S2A). In particular, following low amplitude depolarizing pulses the Off response exhibits a fast decay but not a bump (Figure S2A, $-70mV$). On the other hand, following high amplitude depolarizing pulses the Off response begins with a rising phase and lacks the initial fast decay (Figure S2A, -10 and $+20mV$). The parallel model accounts for feature (*ii*) and also partially for features (*i*) and (*iii*). Specifically, in the Off

response, it accounts for the fast decay, but fails to consistently produce a bump (Figure S2B, inset presenting model simulations of the slow component alone).

Cyclic model

To obtain a bump, similar to the bump observed in the AA-currents, both in the On and the Off responses from a four states cyclic model (scheme S9) the upper arm ($B \rightleftharpoons C \rightleftharpoons E$),without loss of generality, needs to guarantee the formation of a bump during the On responses. In addition, the lower arm ($B \rightleftharpoons D \rightleftharpoons E$) needs to guarantee the formation of a bump during the Off responses. Because each arm is composed of two sequential transitions (scheme S1), transitions $C \rightleftharpoons E$ and $B \rightleftharpoons D$ need to produce most of the measured currents (Eqn S6). Below we show that a prominent bump cannot be achieved concomitantly in the On and the Off currents.

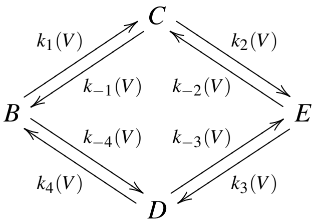

(S9)

For simplicity, let us assume that the gating charge carried by transitions $B \rightleftharpoons C$ and $D \rightleftharpoons E$ is negligible and the gating charge carried by transition $C \rightleftharpoons E$ (q) equal to the gating charge carried by transition $B \rightleftharpoons D$. Under these assumptions the AA-currents (I) of the cyclic model are given by,

$$I = q \times (k_{-4}(V)B + k_2(V)C - k_4(V)D - k_{-2}(V)E) \tag{S10}$$

To obtain the rising phase of the bump in the On response $\dot{I}(t=0)$ need to be positive. Accordingly,

$$\dot{I}(t=0) = q \times \Big(k_{-4}(V)\frac{dB(t=0)}{dt} + k_2(V)\frac{dC(t=0)}{dt} - k_4(V)\frac{dD(t=0)}{dt} - k_{-2}(V)\frac{dE(t=0)}{dt}\Big) > 0 \tag{S11}$$

The kinetic equations that correspond to scheme S9 are given by,

$$\frac{dB}{dt} = -(k_1(V) + k_{-4}(V)) \times B + k_{-1}(V)C + k_4(V)D \tag{S12a}$$

$$\frac{dC}{dt} = -(k_{-1}(V) + k_2(V)) \times C + k_1(V)B + k_{-2}(V)E \tag{S12b}$$

$$\frac{dD}{dt} = -(k_{-3}(V) + k_4(V)) \times D + k_{-4}(V)B + k_3(V)E \tag{S12c}$$

$$R_T = B + C + D + E \tag{S12d}$$

Incorporating Eqn S12 into Eqn S11 provides,

$$\begin{aligned}
\dot{I}(t=0) = & q \times \Bigg(k_4(V) \times \Big(- (k_1(V) + k_{-4}(V)) \times B(t=0) + k_{-1}(V)C(t=0) + k_4(V)D(t=0)\Big) \\
& + k_2(V) \times \Big(- (k_{-1}(V) + k_2(V)) \times C(t=0) + k_1(V)B(t=0) + k_{-2}(V)E(t=0)\Big) \\
& - k_4(V) \times \Big(- (k_{-3}(V) + k_4(V)) \times D(t=0) + k_{-4}(V)B(t=0) + k_3(V)E(t=0)\Big) \\
& - k_{-2}(V) \times \Big(- (k_{-2}(V) + k_3(V)) \times E(t=0) + k_2(V)C(t=0) + k_{-3}(V)D(t=0)\Big)\Bigg) > 0
\end{aligned} \tag{S13}$$

For simplicity, let us further assume that before administration of the depolarizing pulse all the receptors are at state B, i.e., $B(t=0)=R_T$. Hence, $C(t=0)=E(t=0)=D(t=0)=0$. Accordingly,

$$\dot{I}(t=0)=R_T\times q\times\Big(k_{-4}\times(-k_1(V)-k_{-4}(V))+k_2(V)k_1(V)-k_4(V)k_{-4}(V)\Big)>0 \tag{S14}$$

Rearranging Eqn S14 provides the condition needed to be met for a bump to be produced during the On response. Accordingly,

$$k_1(V)k_2(V)>k_{-4}(V)\times(k_1(V)+k_4(V)+k_{-4}(V)) \tag{S15}$$

Similar considerations provide analogous conditions to obtain a bump during the Off response.

$$k_3(V)k_4(V)>k_{-2}(V)\times(k_2(V)+k_3(V)+k_{-2}(V)) \tag{S16}$$

As seen, the conditions in Eqn S15 and S16 will be satisfied for small values of $k_{-2}(V)$ and $k_{-4}(V)$. Moreover, the rate constants $k_{-1}(V)$ and $k_{-3}(V)$ are not included in these conditions. Let us now assign to the cyclic model rate constants that satisfy Eqs. S15 and S16 and account for the time constants of the bump rising phase and decay (text, Figure 2). The value of $k_{-1}(V)$ will be evaluated from the On bump rising phase and $k_{-3}(V)$ will be forced by microscopic reversibility. Figure S3 depicts the On and Off AA-currents, each normalized to its peak amplitude, computed from the cyclic model employing such parameters. As seen, the On response exhibits a prominent bump that resembles the bump observed in the experimental AA-currents recordings. Differently, the Off response exhibits a very fast and small rising phase (Figure S3, inset) that decays slowly. This is because the microscopic reversibility forced a large value for $k_{-3}(V)$. Thus, the rising phase is fast and large fraction of the receptors return to state B via the upper arm which begins with the slow transition $E\longrightarrow C$.

Simplifying the AA-currents model

Following the two simplifying assumptions (see text - Parameters estimation) we notice that at steady state all the receptors that are at state R_1 are also at state C and all the receptors that are at state R_2 are also at state D. Therefore, during the On response the receptors that undergo the transition $C\longrightarrow D$ (with rate constant $k_4(V)$) are the receptors at state C and not at state R_1, i.e., $C-R_1$. Similarly, during the Off response the receptors that undergo the transition $D\longrightarrow C$ (with rate constant $k_{-3}(V)$) are the receptors at state D and not at state R_2, i.e., $D-R_2$. Hence the kinetic equations of the simplified model are given by,

$$\frac{dA}{dt}=-k_1(V)\times A+k_{-1}(V)\times B \tag{S17a}$$

$$R_T=A+B \tag{S17b}$$

$$\frac{dR_1}{dt}=-k_2(V)\times R_1+k_{-2}(V)\times R_2 \tag{S17c}$$

$$R_T=R_1+R_2 \tag{S17d}$$

$$\frac{dC}{dt}=-k_4(V)\times(C-R_1)\qquad \textit{during the On response} \tag{S17e}$$

$$\frac{dC}{dt}=k_{-3}(V)\times(D-R_2)\qquad \textit{during the Off response}$$

$$R_T=C+D \tag{S17f}$$

The AA-currents that are produced by the simplified model are given by,

$$\begin{aligned} I_{On} =& q_1(k_1(V) \times A - k_{-1}(V) \times B) + q_2(k_2(V) \times R_1 - k_{-2}(V) \times R_2) \\ &+ q_3 \times k_4(V) \times (C - R_1) \end{aligned} \tag{S18a}$$

$$\begin{aligned} I_{Off} =& q_1(k_1(V) \times A - k_{-1}(V) \times B) + q_2(k_2(V) \times R_1 - k_{-2}(V) \times R_2) \\ &- q_3 \times k_{-3}(V) \times (D - R_2) \end{aligned} \tag{S18b}$$

Solving Eqn S17 and incorporating into Eqn S18 we obtain,

$$I_{on} = \underbrace{q_1 \times R_T \frac{k_1(V) \times k_{-1}^{HP} - k_{-1}(V) \times k_1^{HP}}{k_1^{HP} + k_{-1}^{HP}} exp(-(k_1(V) + k_{-1}(V))t)}_{transitions\ A \rightleftharpoons B} \tag{S19a}$$

$$+ \underbrace{q_2 \times R_T \times k_2(V) exp(-(k2(V) + k_{-2}(V))t)}_{transitions\ R_1 \rightleftharpoons R_2}$$

$$+ \underbrace{q_3 \times R_T \frac{k_2(V) \times k_4(V)}{k_2(V) + k_{-2}(V) - k_4(V)} (exp(-k_4(V)t) - exp(-(k_2(V) + k_{-2}(V))t))}_{transition\ C \rightarrow D}$$

$$I_{off} = \underbrace{q_1 \times R_T \frac{k_1^{HP} \times k_{-1}(V) - k_{-1}^{HP} \times k_1(V)}{k_1(V) + k_{-1}(V)} exp(-(k_1^{HP} + k_{-1}^{HP})t)}_{transitions\ A \rightleftharpoons B} \tag{S19b}$$

$$+ \underbrace{q_2 \times R_T \times k_{-2}^{HP} exp(-(k2^{HP} + k_{-2}^{HP})t)}_{transitions\ R_1 \rightleftharpoons R_2}$$

$$+ \underbrace{q_3 \times R_T \frac{(\frac{k_{-2}(V)}{k_2(V)+k_{-2}(V)} k_2^{HP} - \frac{k_2(V)}{k_2(V)+k_{-2}(V)} k_{-2}^{HP}) k_{-3}^{HP}}{k_2^{HP} + k_{-2}^{HP} - k_{-3}^{HP}} (exp(-(k_2^{HP} + k_{-2}^{HP})t) - exp(-k_{-3}^{HP} t))}_{transition\ D \rightarrow C}$$

Where $k_{\pm i}^{HP}$ are the rate constants at the holding potential.

Defining,

$$X = q_1 \times R_T \frac{k_1(V) \times k_{-1}^{HP} - k_{-1}(V) \times k_1^{HP}}{k_1^{HP} + k_{-1}^{HP}} \quad \text{or} \tag{S19c}$$

$$X = q_1 \times R_T \frac{k_1^{HP} \times k_{-1}(V) - k_{-1}^{HP} \times k_1(V)}{k_1(V) + k_{-1}(V)}$$

for the On and Off responses respectively

$$Y = q_2 \times R_T \times k_2(V) \tag{S19d}$$

$$Y = q_2 \times R_T \times k_{-2}^{HP}$$

for the On and Off responses respectively

$$Z = q_3 \times R_T \frac{k_2(V) \times k_4(V)}{k_2(V) + k_{-2}(V) - k_4(V)} \quad \text{or} \tag{S19e}$$

$$Z = q_3 \times R_T \frac{(\frac{k_{-2}(V)}{k_2(V)+k_{-2}(V)} k_2^{HP} - \frac{k_2(V)}{k_2(V)+k_{-2}(V)} k_{-2}^{HP}) k_{-3}^{HP}}{k_2^{HP} + k_{-2}^{HP} - k_{-3}^{HP}}$$

for the On and Off responses respectively

$$\lambda_1 = k_1(V) + k_{-1}(V) \tag{S19f}$$

$$\lambda_2 = k_{-3}(V) + k_4(V) \tag{S19g}$$

$$\lambda_3 = k_2(V) + k_{-2}(V) \tag{S19h}$$

Equation S19 assumes the general form of a sum of three exponents. Accordingly,

$$I = \underbrace{X(V)exp(-\lambda_1(V)t)}_{transitions\ A \rightleftharpoons B} + \underbrace{Y(V)exp(-\lambda_3(V)t)}_{transitions\ R_1 \rightleftharpoons R_2}$$
$$+\underbrace{Z(V) \times (exp(-\lambda_2(V)t) - exp(-\lambda_3(V)t))}_{transitions\ C \rightleftharpoons D} \tag{S20}$$

We now examine whether Eqn S20 can faithfully account for the experimental AA-currents. Figure S4A shows that this is indeed the case for three representative depolarizing pulses. Figure S4B depicts the total charge (circles) and the charge carried by each of the transitions, $A \rightleftharpoons B$, $R_1 \rightleftharpoons R_2$ and $C \rightleftharpoons D$ (triangles, diamonds and squares respectively). As seen, the charge carried by the transitions $R_1 \rightleftharpoons R_2$ is insignificant (less than 0.1%) in comparison to the other two transitions. This can be achieved if the value of Y, which is proportional to the effective charge carried by these transitions, is very small or if the value of $e^{-\lambda_3 \times t}$ is very small. The parameters estimation reveals that it is the value of Y which is very small. Therefore, we neglected the contribution of transitions $R_1 \rightleftharpoons R_2$ to the AA-currents. Hence, Eqn S20 is reduced to become,

$$I = \underbrace{X(V)exp(-\lambda_1(V)t)}_{transitions\ A \rightleftharpoons B} + \underbrace{Z(V) \times (exp(-\lambda_2(V)t) - exp(-\lambda_3(V)t))}_{transitions\ C \rightleftharpoons D} \tag{S21}$$

while Eqn 9, the AA-currents of the model in scheme 3 (text), is reduced to become,

$$I = -q_1 \times \frac{dA}{dt} - q_3 \times \frac{dC}{dt} \tag{S22}$$

Figure S4C shows that Eqn S21 maintains the excellent agreement with the experimental results. To see whether the parameters of the fast component can be evaluated from X and λ_1 independently of the slow component and vice versa for the slow component, we expose the two components separately. Figure S4D shows that this is indeed the case. In particular, the fast component fits to the initial fast decay while the slow component fits to the bump.

The final parameters, estimated under the constraint of exponential voltage dependency, are given in Table S1 and the parameters estimated after relaxing this constraint are given in Table S2.

The characteristic features of the transition $R_1 \rightleftharpoons R_2$ are essential

An important assumption in the model is that the transitions $R_1 \rightleftharpoons R_2$ carry insignificant charge in comparison to the transitions $C \rightleftharpoons D$. A surprising result of the model is that in spite of this assumption these transitions strongly depend on voltage (Figure 5 in the text). We argue that the transitions $R_1 \rightleftharpoons R_2$ are likely candidates to relay charge movement to agonist binding affinity. It is therefore important to examine whether the two properties mentioned above are necessary to obtain the AA-currents features.

Figure S5 shows that both properties are indeed essential. The effect of the charge that moves is depicted in Figure S5A. It is seen that when transitions $R_1 \rightleftharpoons R_2$ carry 20 or 40% of the charge that is carried by transitions $C \rightleftharpoons D$ the bump disappears (Figure S5A, green and red lines). When no charge is carried the bump is seen (Figure S5A, blue line). The effect of the voltage dependency of these transitions is examined in Figure S5B and C. Lower voltage dependency is achieved either by increasing the time constant of these transitions at high voltages (Figure S5B, green and red lines) or by decreasing the time constant at low voltages (Figure S5C, green and red lines). It is seen that under the conditions of Figure S5B, in contrast to the experiments and model simulations with the estimated parameters (Figure S5B, blue line), a bump appears in the On response even at $+20mV$, while it disappears at the Off response. Under the conditions of Figure S5C, the bump completely disappears both in the On and the Off responses.

Comparison of average model simulation results to experiments employing unconstraint parameters

Figure S6A depicts the normalized average simulation and experimental AA-currents kinetics employing the unconstraint parameters (Table S2). As seen, the average simulation and experimental AA-currents kinetics match very well (Figure S6A, gray and black lines respectively). The time constants of the fast and the slow components of the AA-currents are depicted in Figure S6B (see text for means to evaluate these time constants). As seen, the experimental and the model time constants match well. The total and the fast and the slow $Q-V$ curves are depicted in Figure S6C. We see that as for the kinetics, the model also describes well the steady-state features of the AA-currents.

References

1. Bezanilla F, Taylor RE (1982) Voltage-dependent gating of sodium channels. In: Culp W, Ochoa J, editors, Abnormal Nerve and Muscle as Abnormal Impulse Generators, New York: Oxford University Press. pp. 62-79.
