# Supplementary figures and images for: New Mechanism for Voltage Induced Charge Movement Revealed in GPCRs - Theory and Experiments
Source: PLoS One. 2010 Jan 22;5(1):e8752. doi: 10.1371/journal.pone.0008752 (PMC2809744; doi:10.1371/journal.pone.0008752)

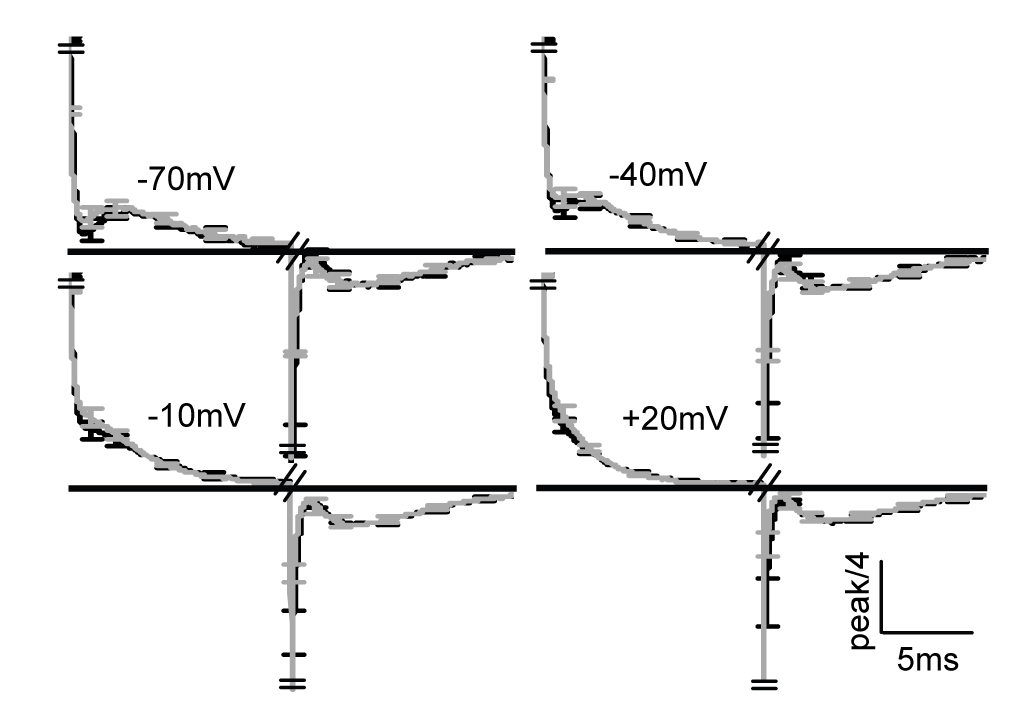

Supplement: Figure S1 — The effect of the ions present in the external solution on the kinetics of the AA-currents. On and Off currents elicited in the m2R expressing oocytes following 40ms depolarizing pulses to the indicated potentials from −120mV. Standard external solution (black lines, see Methods) and when 2mM of CaCl2 was replaced by 2mM of Ba-Acetate (gray lines). The graphs are normalized, each to the peak amplitude of its fast component and are presented as mean ±SD (n = 4–9). (0.06 MB TIF) [file pone.0008752.s002.tif]

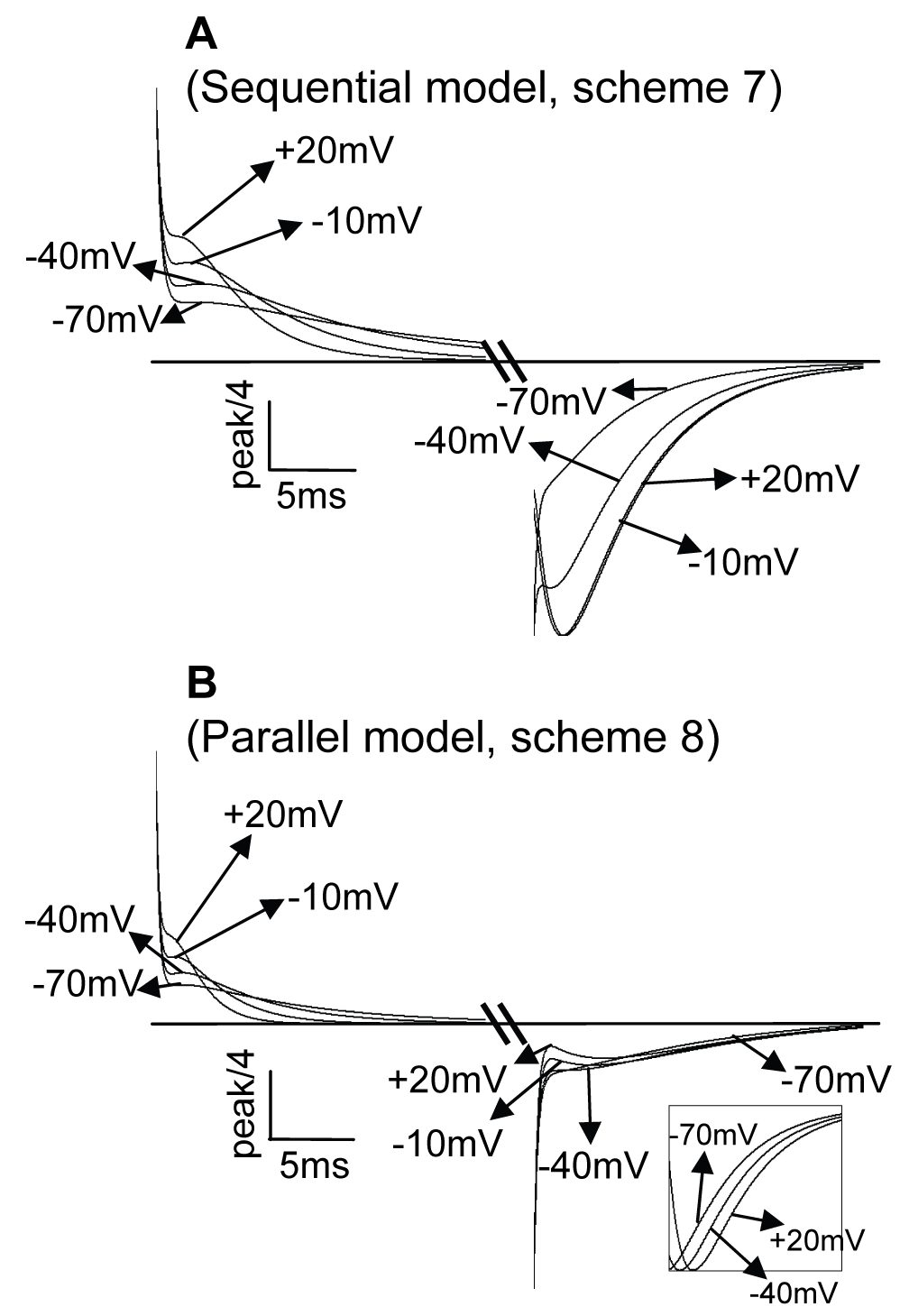

Supplement: Figure S2 — AA-currents predicted by the sequential (scheme 7) and the parallel (scheme 8) models employing the standard protocol to the indicated potentials, (A) and (B), respectively. The Off AA-currents predicted by the slow component of the parallel model (scheme 8) are depicted in the inset of (B). The graphs are normalized, each to the peak amplitude of its fast component. The pulse protocol is presented on top. (0.13 MB TIF) [file pone.0008752.s003.tif]

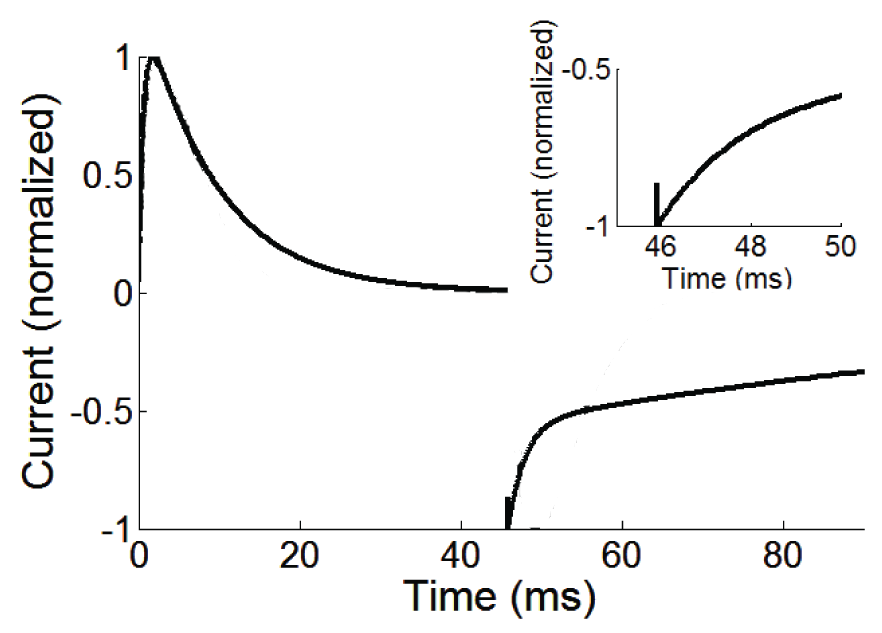

Supplement: Figure S3 — AA-currents predicted by the cyclic model (scheme 9). Computed AA-currents employing 40ms depolarizing pulse to −70mV from a holding potential of −120mV. The model was assigned with parameters that satisfy the conditions in Eqs. 15 and 16 and microscopic reversibility. Inset - the initial phase of the Off response. (0.09 MB TIF) [file pone.0008752.s004.tif]

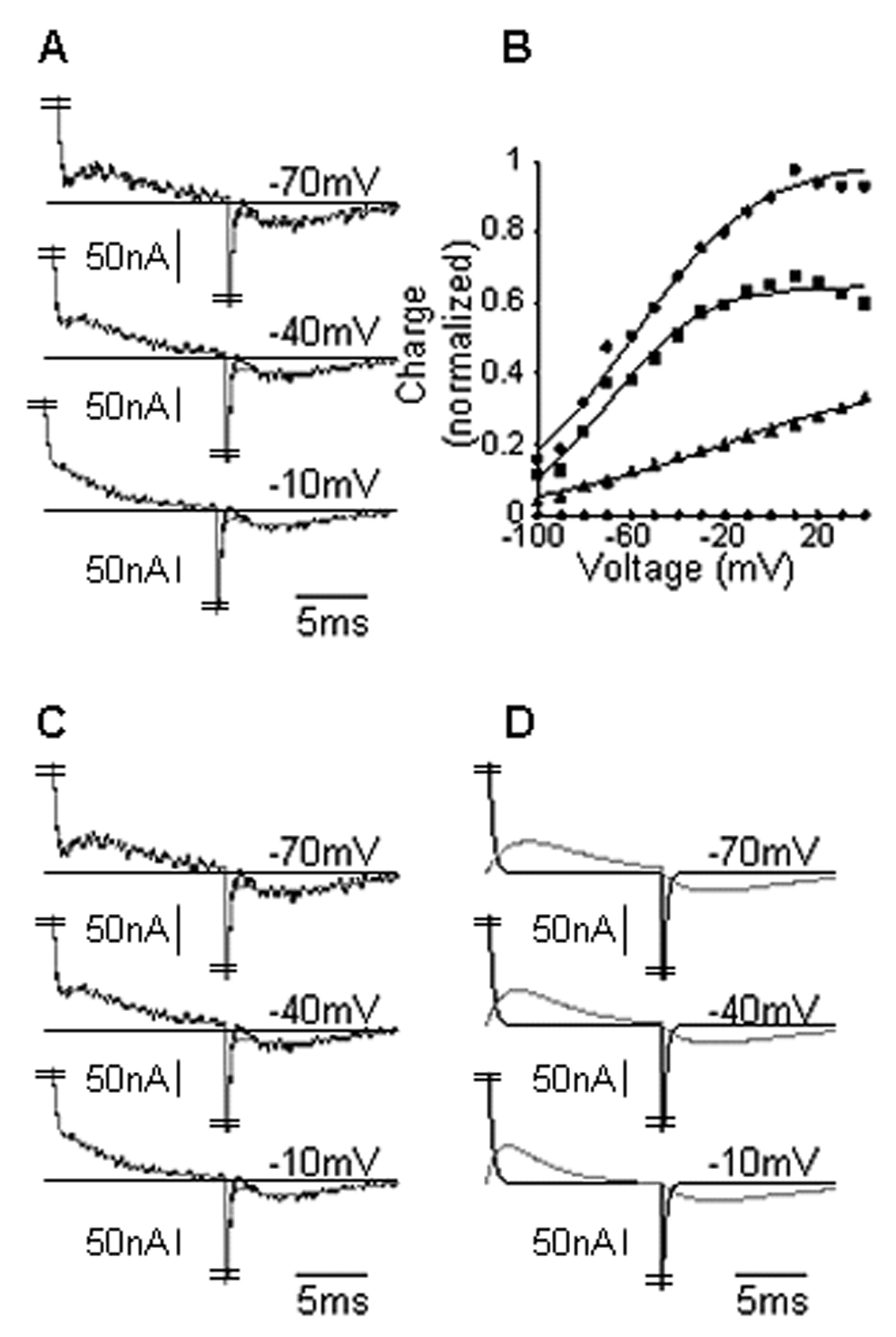

Supplement: Figure S4 — Fitting of Eq. 20 and 21 to the experimental AA-currents recordings. (A) AA-currents recordings from m2R expressing oocytes employing the standard protocol (black lines) superimposed with the three exponential fitting function, Eq. 20 (gray lines). (B) The total charge (circles) and the charge carried by the transitions A↔B (triangles), R1↔R2 (diamonds) and C↔D (squares). (C) AA-currents recordings from m2R expressing oocytes employing the standard protocol (black lines) superimposed with the three exponential fitting function, Eq. 21 (gray lines). (D) Separate plot of the fast and the slow components, solutions were obtained from the same equation (Eq. 21) that was used to fit the experimental results seen in (C). Black lines represent the fast component (Xexp(−λ1t)) and gray lines represent the slow component (Z×(exp(−λ2t)−exp(−λ3t))). (0.26 MB TIF) [file pone.0008752.s005.tif]

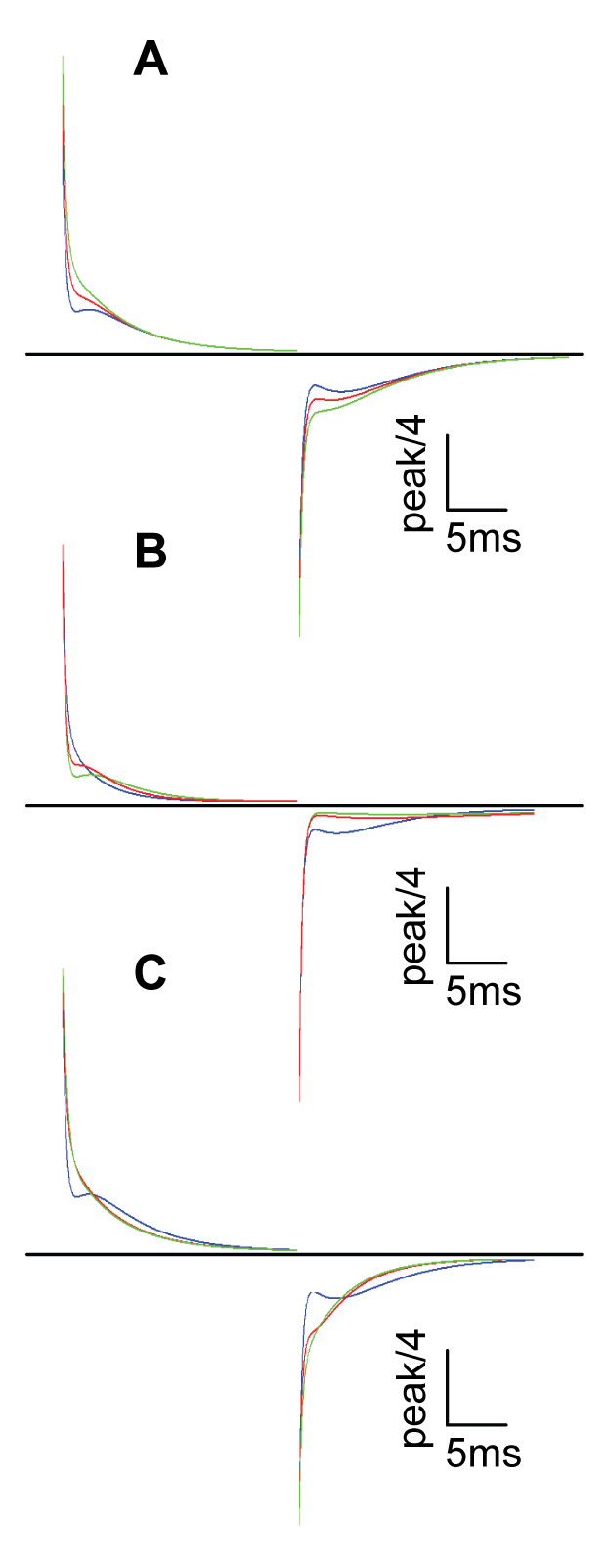

Supplement: Figure S5 — Examining the behavior of transitions R1↔R2. (A) Predicted AA-currents following 40ms depolarizing pulse to −70mV from holding potential of −120mV. The three simulations differ in the value that was assigned to the effective charge carried by transitions R1↔R2. The values were 0, 20 and 40% of the effective charge carried by the transition C↔D (blue, green and red lines respectively). Here and below, the graphs are normalized each to the peak amplitude of its fast component. (B) Predicted AA-currents following 40ms depolarizing pulse to +20mV from holding potential of −120mV. The three simulations differ in the time constant that was assigned to transition R1↔R2 at +20mV. The time constants were: 0.134ms (the time constant that was used throughout, blue line) and 5 and 10 times slower (green and red lines respectively). (C) Predicted AA-currents following 40ms depolarizing pulse to −70mV from holding potential of −120mV. The three simulations differ in the time constant that was assigned to transition R1↔R2 at −70mV. The time constants were: 1.9ms (the time constant that was used throughout, blue line) and 5 and 10 times faster (green and red lines respectively). The model simulation results were obtained using the unconstraint parameters (Table S2). (0.06 MB TIF) [file pone.0008752.s006.tif]

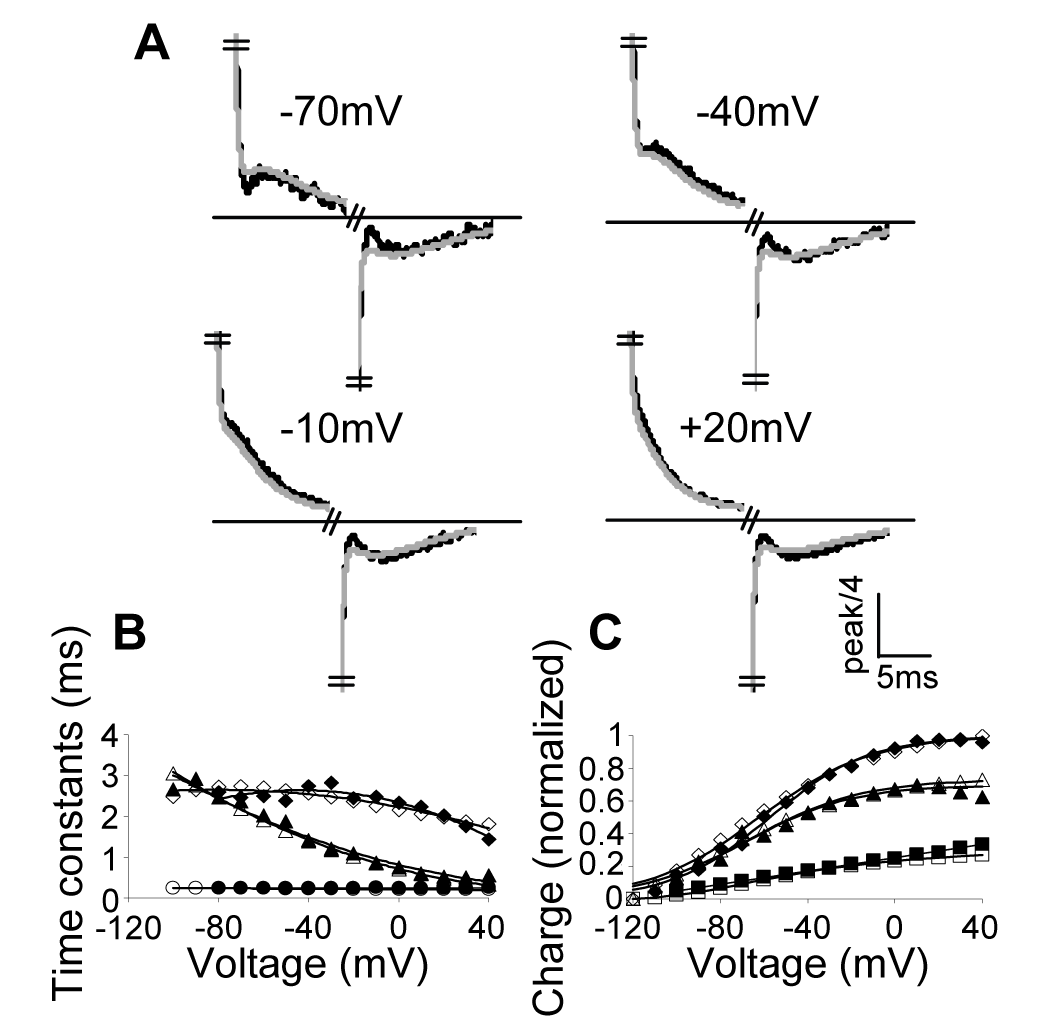

Supplement: Figure S6 — Comparing average (n = 5) simulation and experimental results employing the standard protocol. (A) Kinetics of AA-currents, experiments, black lines, simulations, gray lines. The currents are normalized as describe in Fig. 4 (see text). (B) Time constants of transitions A↔B (circles), R1↔R2 (triangles) and C↔D (diamonds). In all, open symbols correspond to simulations while filled symbols correspond to experiments. (C) Q–V curves of the total charge and the fast and slow components. Diamonds, total Q–V, triangles, Q–V of the slow component and squares, Q–V of the fast component. In all, open symbols correspond to the model and filled symbols to experiments. The parameters were estimated after relaxing the constraint of exponential dependency on membrane potential (Table S2). (0.10 MB TIF) [file pone.0008752.s007.tif]
