# Supplementary material for: New Mechanism for Voltage Induced Charge Movement Revealed in GPCRs - Theory and Experiments
Source: PLoS One. 2010 Jan 22;5(1):e8752. doi: 10.1371/journal.pone.0008752 (PMC2809744; doi:10.1371/journal.pone.0008752)
Supplement: Table S1 — List of the parameters and the standard deviations estimated for the AA-currents model (text, scheme 4). (0.03 MB PDF) [file pone.0008752.s008.pdf]

Parameter	Value	SD
$k_1(s^{-1})$	$4489.5^{(i)} exp(0.00352^{(ii)} V)$	$1729.4^{(i)}$, $0.0033^{(ii)}$
$k_{-1}(s^{-1})$	$634.1^{(i)} exp(-0.00804^{(ii)} V)$	$230.1^{(i)}$, $0.0044^{(ii)}$
$k_2(s^{-1})$	$2307.87^{(i)} exp(0.0282^{(ii)} V)$	$1660.58^{(i)}$, $0.0062^{(ii)}$
$k_{-2}(s^{-1})$	$215.5^{(i)} exp(-0.000049^{(ii)} V)$	$109.48^{(i)}$, $0.00005^{(ii)}$
$k_3(s^{-1})$	$5.82^{(i)} exp(0.00345^{(ii)} V)$	$8.42^{(i)}$, $0.004^{(ii)}$
$k_{-3}(s^{-1})$	$394.98^{(i)} exp(-0.000142^{(ii)} V)$	$129.7^{(i)}$, $0.0002^{(ii)}$
$k_4(s^{-1})$	$395.54^{(i)} exp(0.00345^{(ii)} V)$	$95.8^{(i)}$, $0.004^{(ii)}$
$k_{-4}(s^{-1})$	$0.0033^{(i)} exp(-0.000142^{(ii)} V)$	$0.003^{(i)}$, $0.0002^{(ii)}$
$q_1 \times R_t (C \times M)$	0.525×10^{-9}	0.017×10^{-9}
$q_2 \times R_t (C \times M)$	0.584×10^{-9}	0.014×10^{-9}
