# Supplementary material for: New Mechanism for Voltage Induced Charge Movement Revealed in GPCRs - Theory and Experiments
Source: PLoS One. 2010 Jan 22;5(1):e8752. doi: 10.1371/journal.pone.0008752 (PMC2809744; doi:10.1371/journal.pone.0008752)
Supplement: Table S2 — List of the unconstraint parameters and the standard deviations estimated for the AA-currents model (text, scheme 4). (0.03 MB PDF) [file pone.0008752.s009.pdf]

Parameter	Value	SD
$k_1(s^{-1})$	$\frac{4992.25^{(i)}}{1+exp(0.033^{(ii)}\times(-42.5^{(iii)}-V))}$	$725.8^{(i)}$, $0.01^{(ii)}$, $8.4^{(iii)}$
$k_{-1}(s^{-1})$	$\frac{6109.75^{(i)}}{1+exp(-0.022^{(ii)}\times(-75.6175^{(iii)}-V))}$	$2385.5^{(i)}$, $0.003^{(ii)}$, $18.2^{(iii)}$
$k_2(s^{-1})$	$2172.6^{(i)}exp(0.04^{(ii)}V)$	$1607.5^{(i)}$, $0.006^{(ii)}$
$k_{-2}(s^{-1})$	$224.2^{(i)}exp(-0.00004^{(ii)}V)$	$97.9^{(i)}$, $0.00005^{(ii)}$
$k_3(s^{-1})$	$4.86^{(i)}exp(0.004^{(ii)}V)$	$7.66^{(i)}$, $0.0045^{(ii)}$
$k_{-3}(s^{-1})$	$330.4^{(i)}exp(-0.00006^{(ii)}V)$	$67.48^{(i)}$, $0.00013^{(ii)}$
$k_4(s^{-1})$	$427.3^{(i)}exp(0.004^{(ii)}V)$	$106^{(i)}$, $0.0045^{(ii)}$
$k_{-4}(s^{-1})$	$0.002^{(i)}exp(-0.00006^{(ii)}V)$	$0.0017^{(i)}$, $0.00013^{(ii)}$
$q_1 \times R_t(C \times M)$	0.185×10^{-9}	0.0075×10^{-9}
$q_2 \times R_t(C \times M)$	0.58×10^{-9}	0.014×10^{-9}
